# Interventional treatment for pulmonary venous stenosis due to fibrosing mediastinitis: a case report

**DOI:** 10.3389/fcvm.2025.1633432

**Published:** 2025-08-14

**Authors:** Runfeng Gao, Xulong Yang, Shougang Sun, Hao Hu, Yangyang Yu

**Affiliations:** Department of Cardiovascular Medicine, Second Hospital of Lanzhou University, Lanzhou, China

**Keywords:** fibrosing mediastinitis, pulmonary vein stenosis, angioplasty, stents, contrast-enhanced CT

## Abstract

**Introduction:**

Fibrosing mediastinitis is a rare syndrome caused by the abnormal proliferation of mediastinal fibrous tissue. It often causes superior vena cava syndrome, bronchial stenosis, pulmonary vascular stenosis, etc.

**Patient presentation:**

An elderly male patient presented with intermittent chest tightness, shortness of breath, cough, and large pleural effusion. He was diagnosed with fibrosing mediastinitis through echocardiography, chest CT, and inflammatory indicators. Contrast-enhanced CT and selective angiography showed severe stenosis of multiple branches of bilateral pulmonary veins. The patient is considered to have pulmonary vein stenosis caused by FM, and for further treatment, we performed two interventional therapies at different times. After two operations, the stenosis of bilateral multiple pulmonary veins in this patient has been relieved. Additionally, we also discovered that for pulmonary vein stenosis caused by FM, stent implantation is more effective than balloon dilation.

**Conclusion:**

This case report confirmed that interventional treatment for pulmonary vein stenosis is safe and effective. This study also provides a potential treatment approach for pulmonary vein stenosis resulting from fibrosing mediastinitis.

## Introduction

Fibrosing mediastinitis (FM) is a rare mediastinal fibroproliferative disorder that is associated with chronic inflammation and is closely related to autoimmune diseases involving other systems throughout the body. It is often secondary to infectious diseases such as tuberculosis, atypical mycobacterial infection, nocardiosis, aspergillosis, cryptococcus, and histoplasmosis, and autoimmune diseases such as mediastinal lymphadenitis, including ANCA-associated vasculitis and IgG4-associated multifocal fibrosis. Its main characteristics are the proliferation and sclerosis of mediastinal fibrous tissue caused by various factors, and the fibrous tissue can infiltrate adipose tissue and compress the surrounding tissues, such as airways and blood vessels, causing patients to experience symptoms and signs such as dyspnea, cough, superior vena cava syndrome, and pulmonary hypertension. Mediastinal lymphadenitis due to chronic inflammation involving the mediastinum and immune response can cause allergic reactions and inflammatory changes in the mediastinum, and long-term repeated chronic inflammatory stimulation leads to abnormal fibrous tissue hyperplasia. Compression of pulmonary arteries and pulmonary veins by hyperplasia of hypertrophic mediastinal fibrous tissue can cause pulmonary artery and pulmonary vein stenosis or even occlusion, often causing increased pulmonary artery pressure, and less commonly, pulmonary vein stenosis alone in patients with isolated pulmonary vein stenosis and refractory pleural effusion caused by FM significantly relieves their symptoms after pulmonary venous intervention.

Fibrosing mediastinitis (FM) is a rare mediastinal fibroproliferative disorder that is associated with chronic inflammation and is closely related to autoimmune diseases involving other systems throughout the body. It is often secondary to infectious diseases such as histoplasmosis (1), tuberculosis (2), atypical mycobacterial infection (3), nocardiosis, aspergillosis, cryptococcus (4), and several autoimmune diseases which include ANCA-associated vasculitis, IgG4-related multifocal fibrosis (5), myasthenia gravis (6) and so on. Its main characteristics are the proliferation and sclerosis of mediastinal fibrous tissue caused by various factors, and the fibrous tissue can infiltrate adipose tissue and compress the surrounding tissues, such as airways and blood vessels, causing patients to experience symptoms and signs such as dyspnea, cough, superior vena cava syndrome, and pulmonary hypertension. Mediastinal lymphadenitis due to chronic inflammation involving the mediastinum and immune response can cause allergic reactions and inflammatory changes in the mediastinum, and long-term repeated chronic inflammatory stimulation leads to abnormal fibrous tissue hyperplasia. Excessive mediastinal fibrous tissue compresses the pulmonary blood vessels, causing pulmonary artery and pulmonary vein stenosis or even occlusion (7) and pulmonary hypertension, but pulmonary vein stenosis alone is rare. We reported a patient with isolated pulmonary vein stenosis and refractory pleural effusion due to FM who received pulmonary venous intervention and had significant symptom relief.

## Case description

**Figure 1:**
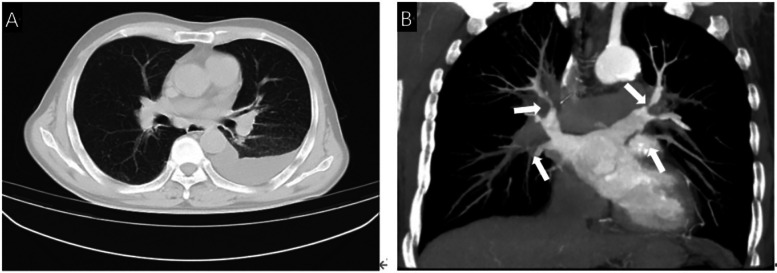
**(A)** Chest CT showed multiple lymph nodes in both hilar areas and left pleural effusion. **(B)** Contrast-enhanced CT showed severe stenosis due to compression of bilateral pulmonary veins by fibrous tissue (White arrow).

A 66-year-old male was admitted to the Second Hospital of Lanzhou University due to chest tightness and shortness of breath with cough. The patient had a history of tuberculosis, 15 years of underground mine work, and occupational exposure to pneumoconiosis. Laboratory tests after admission revealed that the number of neutrophils was 6.59 × 10 × 9/L, the percentage of neutrophils was 0.70, the C-reactive protein level was 26.12 mg/L, the erythrocyte sedimentation rate was 20.0 mm/h, the N-terminal atrial natriuretic peptide level was 75.74 pg/ml, and the pleural effusion sent for testing revealed fluid leakage. There were no obvious abnormalities in coagulation function, liver or kidney function indices, electrolytes, tuberculosis-related examinations, or infectious disease indicators. Cardiac ultrasound revealed that the left ventricle had normal systolic function, mildly decreased diastolic function, a pulmonary artery systolic blood pressure of 55 mmHg, and moderate tricuspid regurgitation. Chest CT without scan imaging revealed multiple nodules and calcifications in both lungs, multiple old lymph nodes in both the hilum and mediastinum, left pleural effusion, and tuberculosis. Contrast-enhanced CT of the pulmonary vein revealed severe pulmonary vein stenosis in both the upper and left lower lobes, and FM was more likely to be considered, as shown in Figure 1.

A 66-year-old male was admitted to the Second Hospital of Lanzhou University due to chest tightness and shortness of breath with cough. The patient had an history of tuberculosis, worked in a mine underground for 15 years, and eventually developed pneumoconiosis due to prolonged exposure to respirable dust particles. Laboratory tests after admission revealed that the number of neutrophils was 6.59 × 10 × 9/L, the percentage of neutrophils was 0.70, the C-reactive protein level was 26.12 mg/L, the erythrocyte sedimentation rate was 20.0 mm/h, the N-terminal atrial natriuretic peptide level was 75.74 pg/ml, and the pleural effusion sent for testing revealed fluid leakage. There were no obvious abnormalities in coagulation function, liver or kidney function indices, electrolytes, tuberculosis-related examinations, or infectious disease indicators. Cardiac ultrasound revealed that the left ventricle had normal systolic function, mildly decreased diastolic function, a pulmonary artery systolic blood pressure of 55 mmHg, and moderate tricuspid regurgitation. Chest CT without scan imaging revealed multiple nodules and calcifications in both lungs, multiple old lymph nodes in both the hilum and mediastinum, left pleural effusion. Contrast-enhanced CT of the pulmonary vein revealed severe pulmonary vein stenosis in both the upper and left lower lobes, and FM was more likely to be considered, as shown in Figure 1.

**Figure 2:**
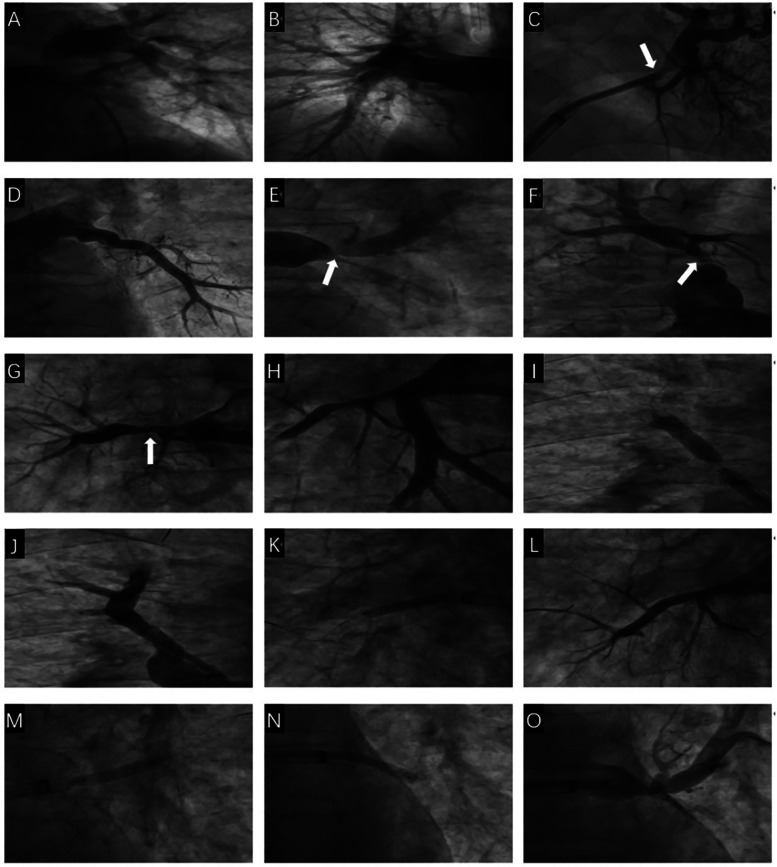
The first pulmonary venous intervention procedure. **(A)** Nonselective angiography showed no obvious stenosis of the left pulmonary artery and its branches. **(B)** Nonselective angiography showed no obvious stenosis of the right pulmonary artery and its branches. **(C)** Selective angiography showed severe stenosis of the opening of the upper and middle branches of the LSPV (White arrow). **(D)** Selective angiography showed no obvious stenosis of the lower branch of the LSPV. **(E)** Selective angiography showed severe stenosis of the two branch openings of the LIPV (White arrow). **(F)** Selective angiography showed severe stenosis of the RSPV opening (White arrow). **(G)** Selective angiography showed severe stenosis of the RMPV opening (White arrow). **(H)** Selective angiography showed no obvious stenosis of the RIPV and its branches. **(I)** A stent is implanted in the RSPV opening. **(J)** Selective angiography showed the stent was fully dilated and there was no residual stenosis in the RSPV. **(K)** A stent was implanted in the RMPV opening. **(L)** Selective angiography showed the stent was fully dilated and there was no residual stenosis in the RMPV. **(M)** LIPV upper branch drug balloon dilation. **(N)** LIPV lower branch drug balloon dilation. **(O)** There is a small amount of residual stenosis after drug balloon dilation of the upper and lower branches of the LIPV.

**Figure 3:**
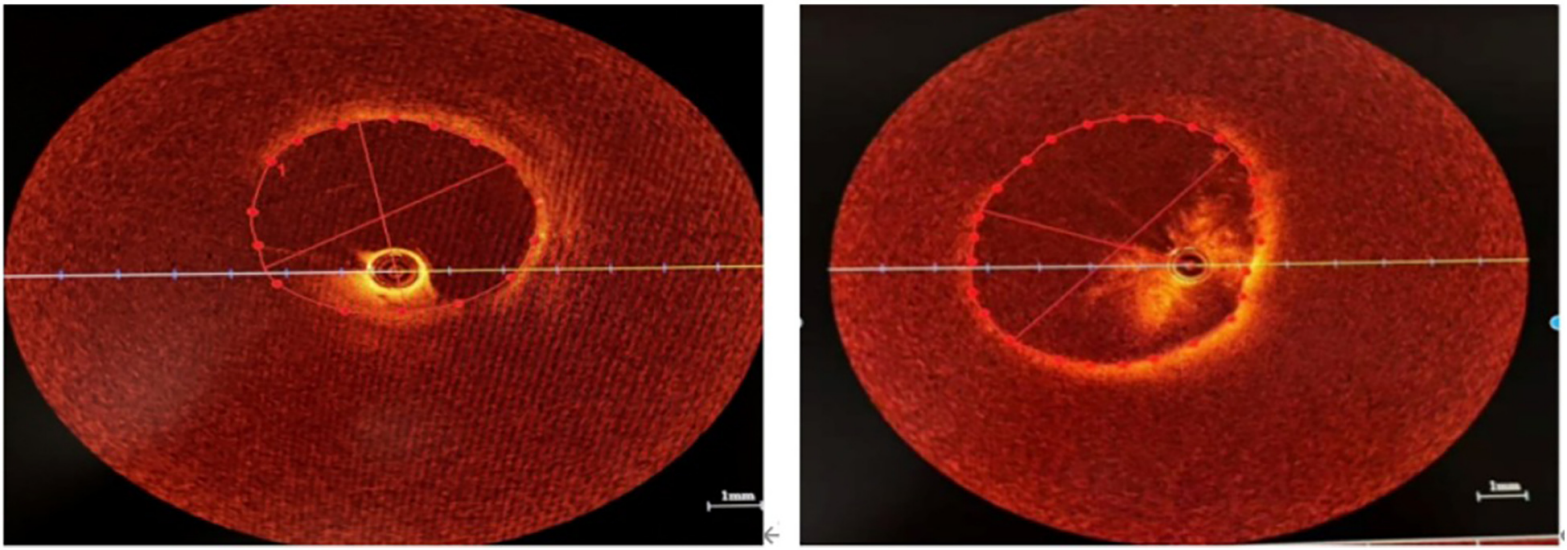
OCT showed that the intimal structure of the venous lumen was intact without plaque and dissection. **(A)** The lumen of the upper branch of the LSPV. **(B)** The lumen of the RSPV.

After providing informed consent for surgery, we first sent a pigtail catheter to the pulmonary artery to measure the systolic pulmonary artery pressure at approximately 45 mmHg and the mean pulmonary artery pressure at approximately 30 mmHg. Nonselective pulmonary angiography with a hyperbaric syringe revealed no occlusion, significant stenosis of the main pulmonary artery trunk and its branches, and pulmonary artery flow grade (PAFG) grade 3 (Figures 2A,B). After puncture of the atrial septum, the steerable sheath (FLEXCATH ADVANCETM, Canada) and JR4.0 guiding catheter (Medtronic, USA) revealed severe stenosis of the opening of the upper branch of the left superior pulmonary vein (LSPV) and opening of the middle branch of the LSPV (Figure 2C), no significant stenosis was found in the lower branch of the LSPV (Figure 2D), severe stenosis of the opening of the upper branch of the left inferior pulmonary vein (LIPV) and opening of the lower branch of the LIPV (Figure 2E), and severe stenosis of the right superior pulmonary vein (RSPV) opening (Figure 2F), as well as the right middle pulmonary vein (RMPV) The JR4.0 guiding catheter was adjusted to the RSPV opening. The Runthrough guidewire (TERUMO, Japan) was sent distally. We used optical coherence tomography (OCT) to carefully observe the luminal structure of the pulmonary vein (Figure 3). After a 3.5 × 15 mm cutting balloon (Boston Scientific, USA) was used for 10 atm dilatation, a 6.0 mm × 24 mm stent (Cordis, Ireland) was implanted into the opening, and angiography revealed good stent expansion and a pulmonary vein flow grade (PVFG). In Grade 3 (Figures 2I,J), the JR4.0 guiding catheter was adjusted to the RMPV opening, the runthrough guidewire was sent distally, OCT was used to measure the diameter and area of the lumen accurately, and the diameter of the lumen was approximately 3.5 mm. A 2.75 × 15 mm cutting balloon (Boston Scientific, USA) was used at 8 atm (1 atm = 760 mmHg) dilatation, followed by implantation of a 3.5 mm × 22 mm stent (Medtronic, USA). Angiography revealed that the well-expended stent was PVFG grade 3 (Figure 2K), the JR4.0 guiding catheter was adjusted to the LIPV, the Runthrough guidewire was sent to the distal end of the upper branch of the LIPV, and a 4.0 mm × 17 mm drug balloon (Medtronic, USA) was used to dilate the lesion at 8 atm. A Runthrough guidewire was sent to the distal end of the lower branch of the LIPV via a 4.0 mm × 20 mm drug balloon (Medtronic, USA), the open lesion was dilated at 6 atm, and angiography revealed a small amount of residual stenosis, PVFG grade 3 (Figures 2M–O). The imaging video is available in the Supplementary Materials. After the operation, the symptoms of chest tightness and shortness of breath were significantly relieved. Follow-up cardiac ultrasound revealed that left ventricular diastolic function was mildly reduced, and the pulmonary artery systolic blood pressure was approximately 27 mmHg. Postoperatively, the patient was given antithrombotic therapy with aspirin and rivaroxaban and discharged from the hospital after removal of the left chest drain.

After signing the informed consent form for interventional treatment, we first sent a pigtail catheter to the pulmonary artery. Nonselective pulmonary angiography revealed no occlusion and significant stenosis of the main pulmonary artery trunk and its branches, and pulmonary artery flow grade (PAFG) grade 3 (Figures 2A,B). After puncture of the atrial septum, the steerable sheath (FLEXCATH ADVANCETM, Canada) and JR4.0 guiding catheter (Medtronic, USA) were delivered to the opening of the pulmonary veins. Selective pulmonary venography was performed separately to show severe stenosis of the opening of the upper branch of the left superior pulmonary vein (LSPV) and opening of the middle branch of the LSPV (Figure 2C), no significant stenosis was found in the lower branch of the LSPV (Figure 2D), severe stenosis of the opening of the upper branch of the left inferior pulmonary vein (LIPV) and the opening of the lower branch of the LIPV (Figure 2E), and severe stenosis of the right superior pulmonary vein (RSPV) opening (Figure 2F), as well as the right middle pulmonary vein (RMPV). Then we adjusted the JR4.0 guiding catheter to the RSPV opening. The Runthrough guidewire (TERUMO, Japan) was sent to end of the RSPV. We used optical coherence tomography (OCT) to carefully observe the luminal structure of the pulmonary vein (Figure 3). After a 3.5mm × 15 mm cutting balloon (Boston Scientific, USA) was used for 10 atm dilatation, a 6.0 mm × 24 mm stent (Cordis, Ireland) was implanted into the opening of the RSPV, and angiography revealed good stent expansion and a pulmonary vein flow grade (PVFG) in Grade 3 (Figures 2I,J). Then the JR4.0 guiding catheter was adjusted to the RMPV opening, the Runthrough guidewire was sent to distal, OCT was used to measure the diameter and area of the lumen accurately, and the diameter of the lumen was approximately 3.5 mm. A 2.75 mm × 15 mm cutting balloon (Boston Scientific, USA) was used at 8 atm (1 atm = 760 mmHg) dilatation, followed by implantation of a 3.5 mm × 22 mm stent (Medtronic, USA). Angiography revealed that the well-expended stent was PVFG grade 3 (Figure 2K). Then we adjusted the guiding catheter to the LIPV, sent the Runthrough guidewire to the distal end of the upper branch of the LIPV, and a 4.0 mm × 17 mm drug balloon (Medtronic, USA) was used to dilate the lesion at 8 atm. Eventually, we sent the Runthrough guidewire to the distal end of the lower branch of the LIPV, then a 4.0 mm × 20 mm drug balloon (Medtronic, USA) was dilated at 6 atm in the opening, and angiography revealed a small amount of residual stenosis, PVFG grade 3 (Figures 2M–O). The imaging video is available in the Supplementary Materials. We used a right cardiac catheter to measure the PAP of about 46/24 mmHg, and the mean PAP was 31 mmHg. After the operation, the symptoms of chest tightness and shortness of breath were significantly relieved. Follow-up cardiac ultrasound revealed that left ventricular diastolic function was mildly reduced, and the pulmonary artery systolic blood pressure was approximately 27 mmHg. Postoperatively, the patient was given antithrombotic therapy with aspirin and rivaroxaban and discharged from the hospital after removal of the left chest drain.

**Figure 4:**
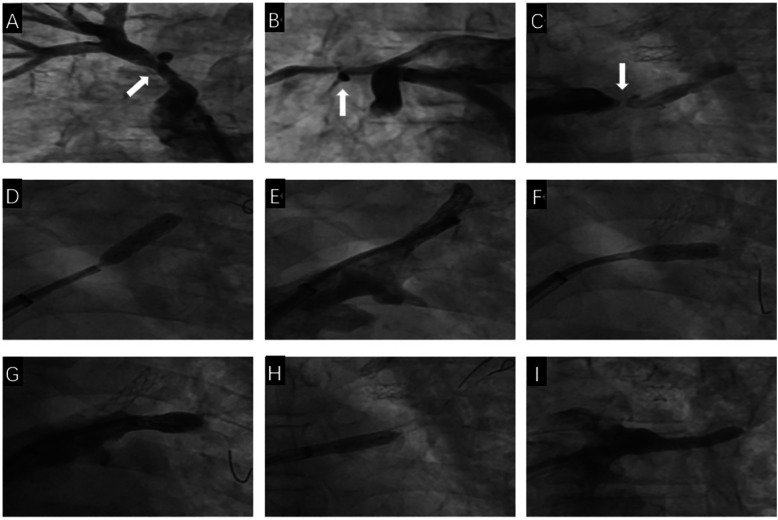
The second pulmonary venous intervention process. **(A)** Selective angiography shows mild restenosis in the stent of the RSPV (White arrow). **(B)** Nonselective contrast shows mild restenosis in the stent of the RMPV (White arrow). **(C)** Selective angiography shows severe stenosis at the opening of the upper branch of the LIPV and occlusion of the lower branch of the LIPV (White arrow). **(D)** A stent is implanted at the opening of the upper branch of the LSPV. **(E)** Selective angiography after stent implantation in the upper branch of the LSPV shows full expansion of the stent, with no residual stenosis. **(F)** A stent is implanted at the opening of the middle branch of the LSPV. **(G)** Selective angiography after stent implantation in the middle branch of the LSPV shows mild residual stenosis. **(H)** A stent is implanted at the opening of the upper branch of the LIPV. **(I)** Selective angiography after stent implantation in the upper branch of the LIPV shows full expansion of the stent, with no residual stenosis.

One month later, the patient was readmitted to the hospital due to prolonged accumulation of pleural effusion. Reexamination via pulmonary venography after admission revealed mild restenosis of the RSPV opening stent (Figure 4A), mild restenosis of the right middle pulmonary vein opening stent, no abnormalities in the RIPV (Figure 4B), severe stenosis of the opening of the upper branch of the LIPV, and occlusion of the lower branch of the LIPV (Figure 4C). A steerable sheath (FLEXCATH ADVANCETM Canada) and JR4.0 guiding catheter (Medtronic, USA) was fed into the upper branch of the LSPV, and the Runthrough guidewire (TERUMO, Japan) to distal, a 2.5 × 15 mm balloon (Medtronic, USA) was applied to the LSPV opening lesion and dilatated at 12 atm. Then, a 6.0 × 18 mm stent was implanted (Cordis, Ireland), with no significant residual stenosis or dissection on contrast, PVFG grade 3 (Figures 4D,E). Then, the steerable sheath and guiding catheter were adjusted to the opening of the middle branch of the LSPV, the Runthrough guidewire was sent to the distal end, and a 2.5 × 15 mm balloon (Medtronic, United States) to the LSPV intermediate branch opening dilatated at 10 atm and implanted with a 7.0 × 15 mm stent (Cordis, Ireland), with a distinct residual stenosis. The imaging video is available in the Supplementary Materials. A chest CT scan 6 months after surgery showed a small amount of fluid accumulation on the left side, and symptoms such as shortness of breath were significantly alleviated.

One month later, the patient was readmitted to the hospital due to prolonged accumulation of pleural effusion. Reexamination via pulmonary venography revealed mild restenosis of the RSPV opening stent (Figure 4A), mild restenosis of the right middle pulmonary vein opening stent, no abnormalities in the RIPV (Figure 4B), severe stenosis of the opening of the upper branch of the LIPV, and occlusion of the lower branch of the LIPV (Figure 4C). We sent the steerable sheath (FLEXCATH ADVANCETM Canada) and the JR4.0 guiding catheter (Medtronic, USA) into the upper branch of the LSPV, and sent the Runthrough guidewire (TERUMO, Japan) to distal, a 6.0 mm × 18 mm stent was implanted (Cordis, Ireland) after a 2.5 mm × 15 mm balloon (Medtronic, USA) was dilatated at 12 atm in the opening of the LSPV, selective angiography showed no significant residual stenosis, PVFG grade 3 (Figures 4D,E). Then, the steerable sheath and guiding catheter were adjusted to the opening of the middle branch of the LSPV, we implanted with a 7.0 mm × 15 mm stent (Cordis, Ireland) after a 2.5 mm × 15 mm balloon (Medtronic, United States) was dilatated at 10 atm in opening of the LSPV intermediate branch, selective angiography showed a distinct residual stenosis, PVFG grade 3 (Figures 4F,G). In the end, we adjusted the steerable sheath and guiding catheter to the upper branch of the LIPV, a 6.0 mm × 15 mm stent was implanted (Cordis, Ireland) after a 2.5 mm × 15 mm balloon (Medtronic, USA) was dilatated at 10 atm in the opening of the upper branch of the LIPV, selective angiography showed no residual stenosis, PVFG grade 3 (Figures 4H,I). The imaging video is available in the Supplementary Materials. Similarly, before and after the second operation, we used a right cardiac catheter to measure the mean PAP of 34 mmHg and 32 mmHg, respectively. A chest CT scan 6 months after operation showed a small amount of fluid accumulation on the left side, and symptoms such as shortness of breath were significantly alleviated.

## Discussion

In our case, the patient had a history of tuberculosis in the past, had a history of coal mining occupation for many years, had significantly elevated CRP and ESR on admission, and showed significant fibrous tissue hyperplasia of mediastinal and bilateral hilar structures on contrast-enhanced CT; additionally, the lesions were diffusely distributed, meeting the diagnostic criteria for FM. During FM, mediastinal fibrous hyperplasia can cause pulmonary vascular disease, and narrowing due to fibrous compression has been shown to often involve the pulmonary arteries (8). However, pulmonary artery stenosis was not obvious in this patient, and pulmonary artery pressure was slightly elevated, mainly manifesting as severe stenosis of multiple bilateral pulmonary veins and refractory pleural effusion, which is relatively rare in clinical practice. Treatment of pulmonary vein stenosis includes medications, interventional procedures, surgery, and even lung transplantation (9). Drug treatment mainly includes steroids, pulmonary vasodilators, immunosuppressants, angiotensin II receptor blockers, etc. (10–12). In the past, several small studies have shown that the above drugs can alleviate the symptoms of patients to a certain extent, but few large studies have confirmed their efficacy and safety, and they cannot fundamentally solve this problem. Similarly, for bilateral multivessel pulmonary vein stenosis caused by FM, some investigators have chosen to excise the thickened pulmonary vein wall under the protection of extracorporeal membrane oxygenation (ECMO) and reconstruct the pulmonary veins with a bovine pericardial patch (13), but thoracotomy is at greater risk of trauma and infection. At present, interventional therapy is the preferred treatment for pulmonary vascular stenosis or occlusion caused by FM.

In our case report, we described a patient with severe pulmonary venous stenosis caused by FM with intractable pleural effusion. We mainly found that: (1) Intractable pleural effusion is extremely rare in patients with FM that causes pulmonary vein stenosis alone, the preferred treatment is interventional therapy; (2) In our interventional process, OCT was innovatively used to guide the implantation of stents, this not only improved the effect of the operation but also avoided damaging the delicate pulmonary veins; (3) Through the comparison of the two interventional procedures, we concluded that stent implantation is more effective than balloon dilation for pulmonary vein stenosis caused by FM. In our case, the patient had a history of tuberculosis in the past, had a history of coal mining occupation for many years, had significantly elevated CRP and ESR on admission, and showed significant fibrous tissue hyperplasia of mediastinal and bilateral hilar structures on contrast-enhanced CT, additionally, the lesions were diffusely distributed, meeting the diagnostic criteria for FM. During FM, mediastinal fibrous hyperplasia can cause pulmonary vascular disease, and stenosis due to fibrous compression has been shown to often involve the pulmonary arteries (8). However, pulmonary artery stenosis was not obvious in this patient and pulmonary artery pressure was slightly elevated, mainly manifesting as severe stenosis of multiple bilateral pulmonary veins and intractable pleural effusion, which is relatively rare in clinical practice. Treatment of pulmonary vein stenosis includes medications, interventional procedures, surgery, and even lung transplantation (9). Drug treatment mainly includes steroids, pulmonary vasodilators, immunosuppressants, angiotensin II receptor blockers, etc. (10–12). In the past, several studies have shown that the above drugs can alleviate the symptoms of patients to a certain extent, but few large studies have confirmed their efficacy and safety, and they cannot fundamentally solve this problem. Similarly, for bilateral multivessel pulmonary vein stenosis caused by FM, some investigators have chosen to excise the thickened pulmonary vein wall under the protection of extracorporeal membrane oxygenation (ECMO) and reconstruct the pulmonary veins with a bovine pericardial patch (13), but thoracotomy is at greater risk of trauma and infection. At present, interventional therapy is the preferred treatment for pulmonary vascular stenosis or occlusion caused by FM.

Contrast-enhanced CT is the preferred method for estimating the degree and severity of pulmonary vascular involvement before interventional therapy by identifying vascular lesions before surgery (14). However, CT has a limited role in pulmonary venous intervention. Owing to the thin membrane of the vascular wall, it is necessary to avoid damage to the venous wall as much as possible during the balloon expansion and stent release process, and the maximum diameter of the stent implanted is limited by the cross-sectional area of the venous stenosis. The normal venous segment around the stenosis has a relatively high level of compliance. Therefore, accurately measuring the diameter and area of the target pulmonary vein lumen before balloon dilation and stent implantation is important, and we innovatively use OCT to evaluate pulmonary vein vessels before stent implantation. Because of the high resolution, some researchers have used OCT to evaluate the intraluminal anatomy of blood vessels in interventions for pulmonary vein stenosis in children, which has improved the safety and effectiveness of the operation (15). However, the use of OCT in FM-related pulmonary venous intervention has not been reported in the literature. During pulmonary venography, we observed the structure of the pulmonary vein wall via OCT and revealed that the pulmonary venous intima was smooth without lipid deposition or atherosclerosis and that the patient's mediatic systolic function was good, confirming that the patient's pulmonary vein stenosis was caused by the proliferation and compression of mediastinal fibrous tissue distributed outside the vascular lumen. More importantly, we used OCT to accurately measure the lumen diameters of the RMPV and RSPV at 3.5 mm and 5.15 mm, so 3.5 mm × 22 mm stents and 6.0 mm × 24 mm stents were implanted, respectively. This technique helps us select the appropriate size balloon and stent to ensure good adhesion after stent release, improve the surgical outcome, and avoid damage to the pulmonary vein wall structure.

Contrast-enhanced CT is the preferred method for estimating the degree and severity of pulmonary vascular involvement before interventional therapy (14). However, owing to the thin membrane of the pulmonary vein, it is necessary to avoid damage to the venous wall as much as possible during the balloon expansion and stent release process, and the maximum diameter of the stent implanted is limited by the cross-sectional area of the venous stenosis. The normal venous segment around the stenosis has a relatively high level of compliance. Therefore, accurately measuring the diameter and area of the target pulmonary vein lumen before balloon dilation and stent implantation is important, and we innovatively use OCT to evaluate pulmonary vein vessels before stent implantation. Because of the high resolution, some researchers have used OCT to evaluate the intraluminal anatomy of blood vessels in interventions for pulmonary vein stenosis in children, which has improved the safety and effectiveness of the operation (15). However, the use of OCT in FM-related pulmonary venous intervention has not been reported in the literature. During pulmonary venography, we observed the structure of the pulmonary vein wall via OCT and revealed that the pulmonary venous intima was smooth without lipid deposition or atherosclerosis and mediatic systolic function was well, confirming that the patient's pulmonary vein stenosis was caused by the proliferation and compression of mediastinal fibrous tissue distributed outside the vascular lumen. More importantly, we used OCT to accurately measure the lumen diameters of the RMPV and RSPV at 3.5 mm and 5.15 mm, so 3.5 mm × 22 mm stents and 6.0 mm × 24 mm stents were implanted, respectively. The new type technique helped us select the appropriate size balloon and stent to ensure good adhesion after stent release, improve the operation outcome, and avoid damage to the pulmonary veins wall structure.

In our case, we found that, for pulmonary vein stenosis caused by FM, the implantation of stents was superior to balloon dilation, and during the patient's first intervention, we performed pulmonary venography and found that the patient had multiple severe stenoses of the bilateral pulmonary veins. Due to the lack of previous experience in pulmonary vein stenosis intervention, to ensure the safety of the operation and reduce the operation time, we decided to treat the stenosis in batches after the evaluation by OCT. A stent was then implanted in the RMPV, and a stent in the RSPV and the upper and lower branches of the LIPV were dilated with a drug balloon, and the patient's symptoms were relieved after surgery. The second pulmonary venography revealed that the opening of the upper branch of the LIPV was severely narrowed after implantation of the drug balloon, the lower branch was blocked, and mild restenosis of the vascular lumen of the RSPV and RMPV was observed after implantation with stents. Considering that pulmonary vein stenosis caused by mediastinal fibrous tissue compression has a limited effect on balloon dilation and that it is necessary to use the rigid structure provided by the stent to resist external pressure, we implanted stents in both branches of the LSPV during the second intervention, and the patient's symptoms, such as dyspnea, significantly improved after the operation. Additionally, the chest CT scan was reexamined 6 months later, and the incidence of pleural effusion was significantly lower than before. The use of cutting balloons and drug balloons to dilate pulmonary vein stenosis during pulmonary venous intervention is effective, but the long-term efficacy is poor (16, 17). In the interventional treatment of pulmonary vein stenosis, the restenosis rate is significantly lower with stenting than with balloon dilation (18, 19). This is consistent with the conclusions we have reached. In addition, when we reviewed the angiography results, we found that the patient had restenosis of the RMPV stent, which was deformed due to continuous compression of the mediastinal fibrous tissue, which was completely different from the mechanism of restenosis after coronary artery stent implantation, which was studied previously (20). It has been suggested that the implantation of drug-eluting stents during initial pulmonary venous intervention can effectively improve pulmonary vein stenosis, but the long-term benefit for patients is not good. Therefore, the patient may need to be admitted to the hospital for regular follow-up pulmonary venography, and if restenosis is found in the implanted stent, in-stent balloon dilation can be performed to maintain the surgical effect; in the same way, an elective reattempt to open the occluded left lower pulmonary vein may help to further improve the patient's prognosis.

In our case, we concluded that, for pulmonary vein stenosis caused by FM, the implantation of stents was superior to balloon dilation, and during the patient's first intervention, we performed pulmonary venography and found that the patient had multiple severe stenoses of the bilateral pulmonary veins. Due to the lack of previous experience in pulmonary vein stenosis intervention, and to ensure the safety of the operation and reduce the operation time, we decided to perform the operation in batches. A stent was then implanted in the RMPV, and a stent in the RSPV and the upper and lower branches of the LIPV were dilated with a drug balloon, and the patient's symptoms were relieved after procedure. The second pulmonary venography revealed that the opening of the upper branch of the LIPV was severely narrowed after implantation of the drug balloon, the lower branch was blocked, and mild restenosis of the vascular lumen of the RSPV and RMPV was observed after implantation with stents. Considering that pulmonary vein stenosis caused by mediastinal fibrous tissue compression has a limited effect on balloon dilation and that it is necessary to use the rigid structure provided by the stent to resist external pressure, we implanted stents in both branches of the LSPV during the second intervention, and the patient's symptoms, such as dyspnea, significantly improved after the operation. Additionally, the chest CT scan was reexamined 6 months later, and the incidence of pleural effusion was significantly lower than before. The use of cutting balloons and drug balloons to dilate pulmonary vein stenosis during pulmonary venous intervention is effective, but the long-term efficacy is poor (16, 17). In the interventional treatment of pulmonary vein stenosis, the restenosis rate is significantly lower with stenting than with balloon dilation (18, 19). This is consistent with the conclusions we have reached. In addition, when we reviewed the angiography results, we found that the patient had restenosis of the RMPV stent, which was deformed due to continuous compression of the mediastinal fibrous tissue, which was completely different from the mechanism of restenosis after coronary artery stent implantation, which was studied previously (20). It has been suggested that the implantation of drug-eluting stents during initial pulmonary venous intervention can effectively alleviate pulmonary vein stenosis, but the long-term benefit for patients is not well. Therefore, the patient may need to be admitted to the hospital for regular follow-up, and if restenosis is found in the implanted stent, in-stent balloon dilation can be performed to maintain the surgical effect, in the same way, an elective reattempt to open the occluded left lower pulmonary vein may help to further improve the patient's prognosis.

## Conclusion

For pulmonary vein stenosis caused by FM, the effect of drug treatment is limited; interventional therapy is the preferred treatment, and the ability of stent implantation to relieve pulmonary vein stenosis is better than that of balloon dilation. However, its safety and efficacy still need to be demonstrated by a larger number of studies. Patients need to monitor their condition for a long time after receiving interventional therapy, and if necessary, they can be reintervened on again or multiple times regularly to improve their quality of life and long-term survival.

For pulmonary vein stenosis caused by FM, the effect of drug treatment is limited; interventional therapy is the preferred treatment, and the ability of stent implantation to alleviate pulmonary vein stenosis is better than balloon dilation. However, its safety and efficacy still need to be demonstrated by a larger number of studies. Patients need to monitor their condition for a long time after receiving interventional therapy, and if necessary, they can be reintervened on again or many times regularly to improve their quality of life and long-term survival.

## Data Availability

The original contributions presented in the study are included in the article/Supplementary Material, further inquiries can be directed to the corresponding author.
